# Postnatal and long-term outcomes after in utero exposure to RAAS inhibitors: cohort study based on German claims data

**DOI:** 10.1007/s00467-025-07101-9

**Published:** 2025-12-17

**Authors:** Tania Schink, Malte Braitmaier, Katarina Dathe, Ulrike Haug, Christof Schaefer, Kathrin Thöne, Marlies Onken

**Affiliations:** 1 Department of Clinical Epidemiology, Leibniz Institute for Prevention Research and Epidemiology – BIPS, Bremen, Germany; 2 Department of Statistical Methods in Epidemiology, Leibniz Institute for Prevention Research and Epidemiology – BIPS, Bremen, Germany; 3 Pharmakovigilanz- und Beratungszentrum für Embryonaltoxikologie, Institut für Klinische Pharmakologie und Toxikologie, Charité – Universitätsmedizin Berlin, corporate member of Freie Universität Berlin, Humboldt-Universität zu Berlin, and Berlin Institute of Health, Berlin, Germany; 4 Faculty of Human and Health Sciences, University of Bremen, Bremen, Germany; 5 Techniker Krankenkasse, Hamburg, Germany

**Keywords:** Renin–angiotensin–aldosterone system inhibitors, Angiotensin-converting enzyme (ACE) inhibitors, Angiotensin II type 1 receptor blockers (ARBs), Pregnancy, Drug-related side effects and adverse reactions, Long-term outcome

## Abstract

**Background:**

Although use of inhibitors of the renin–angiotensin–aldosterone system (RAAS-I) is contraindicated in the second and third trimesters of pregnancy, a relevant number of pregnancies is still exposed. Fetopathy in children exposed after gestational week (GW) 20 is well described, but data on long-term outcomes are scarce. Our study aims to describe postnatal and long-term outcomes after fetal exposure to RAAS-I.

**Methods:**

We included all pregnancies in the German Pharmacoepidemiological Research Database GePaRD (claims data; 20% of the total German population) with exposure to RAAS-I or the antihypertensives recommended during pregnancy, i.e., metoprolol or methyldopa (HYP) after GW 20. We assessed diagnoses characteristic of RAAS-I-related fetopathy in the first 180 days after birth and examined long-term outcomes of children with and without neonatal fetopathy, especially hypertension and kidney disease.

**Results:**

Overall, we identified 203 live born children exposed to RAAS-I, of whom 61 were exposed to angiotensin II receptor blockers (ARBs), and 29,674 live born children exposed to HYP. Diagnoses consistent with RAAS-I-related fetopathy were seen in eight of the RAAS-I exposed newborns (3.9%) and in seven of the 61 ARB-exposed newborns (11.5%). Median follow-up in children without fetopathy was 4.0 years in both exposure groups. Among non-fetopathy children exposed to RAAS-I, three (1.5%) were diagnosed with hypertension or received antihypertensive prescriptions, compared to 176 children (0.6%) exposed to HYP.

**Conclusions:**

Risk of fetopathy is higher after fetal exposure to ARBs than to angiotensin-converting enzyme inhibitors. In a small proportion of children, sequelae of fetal RAAS-I exposure might only manifest in the years following birth.

**Graphical Abstract:**

**Figure:**
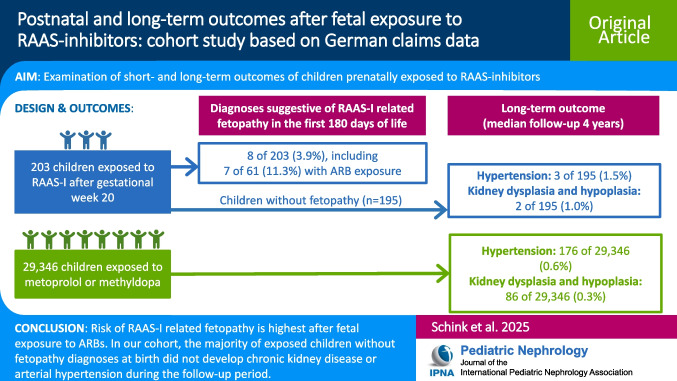
A higher resolution version of the Graphical abstract is available as Supplementary information

**Supplementary Information:**

The online version contains supplementary material available at 10.1007/s00467-025-07101-9.

## Introduction

Inhibitors of the renin–angiotensin–aldosterone system (RAAS-I), i.e., renin inhibitors, angiotensin-converting-enzyme (ACE) inhibitors, and angiotensin II receptor blockers (ARBs), are routinely used in the treatment of arterial hypertension, also in women of childbearing age [1]. While data on pregnancy outcome after first trimester exposure to RAAS-I are conflicting [2], the harmful effects of RAAS-I exposure in the second half of pregnancy are well established: It has been shown that treatment with RAAS-I may compromise fetal kidney function and urine production from around gestational week (GW) 20, resulting in amniotic fluid reduction [1]. Apart from oligo- and anhydramnios, neonatal kidney failure, lung hypoplasia, hypocalvaria/widened skull sutures, joint contractures, vena cava thrombosis, and fetal or neonatal death have been reported [3–6]. Therefore, use of RAAS-I is contraindicated in the second and third trimester of pregnancy.

A recent study from the USA analyzed utilization patterns for antihypertensives among pregnant patients with pre-existing hypertension and found a significant number of RAAS-I-exposed pregnancies [1]: Prior to pregnancy, the prevalence of RAAS-I use was 19.5% (*n* = 2537/12,978) for pregnancies ending in a live birth and 24.7% (*n* = 1875/7,598) among those with pregnancy loss. During pregnancy, the majority of women switched to a more appropriate antihypertensive medication. However, 0.5% (*n* = 68/12,881) and 0.6% (*n* = 71/12,881) of pregnancies ending in live births were still exposed to ACE inhibitors and ARBs, respectively, in the third trimester.

This study shows that, despite the contraindication, exposure to RAAS-I after GW 20 still occurs. It is therefore important to consider possible effects on the health of these children, not only regarding the known and well-described fetopathy but also regarding longer-term effects. However, data on long-term outcomes of prenatally exposed children are scarce [7]. Laube et al. reported on three prenatally exposed children from pregnancies without oligohydramnios who presented with transient anuria after delivery [8]. Their kidney function recovered within 3 months, but at school age, two of them presented with progressive kidney impairment, one also with arterial hypertension. Plazanet et al. reported that kidney damage (interstitial fibrosis and cortical cysts but not renal tubular dysgenesis) was detectable in autopsied fetuses/infants even in cases where oligohydramnios had been reversible after RAAS-I discontinuation [9]. It has therefore been hypothesized that prenatal RAAS-I exposure might impair kidney function and cause arterial hypertension with a time delay. To our knowledge, no study has investigated whether children exposed to RAAS-I in utero without diagnosed fetopathy in the first 6 months of life are at risk of developing arterial hypertension or chronic kidney disease later in life. Existing case series primarily describe the clinical course of infants born with symptoms of RAAS-I-related fetopathy [10–12] or provide follow-up for only a short postnatal period [4].

To shed further light on the health of children with prenatal RAAS-I exposure, we aimed (i) to assess the proportion of exposed children with potential manifestations of RAAS-I-related fetopathy, (ii) to assess the postnatal clinical course of children with RAAS-I-related fetopathy and, to address the aforementioned knowledge gap, (iii) to describe the long-term outcomes of prenatally exposed children.

## Methods

### Data source

We used the German Pharmacoepidemiological Research Database (GePaRD) for this study, which is based on claims data from four statutory health insurance providers in Germany and currently includes information on approximately 25 million persons who have been insured with one of the participating providers since 2004 or later. In addition to demographic data, GePaRD contains information on drug dispensings as well as outpatient (i.e., from general practitioners and specialists) and inpatient services and diagnoses. Per data year, there is information on approximately 20% of the general population and all geographical regions of Germany are represented [13].

For research on drug utilization and safety during pregnancy, algorithms to identify and classify pregnancy outcomes [14, 15], estimate pregnancy onset [16], and link mothers with their children [17] have been developed for GePaRD. In brief, pregnancy onset is estimated using the expected delivery date, which is determined based on the last menstrual period (LMP) or early ultrasound. This information is available for about 80% of pregnancies in GePaRD. If no (plausible) expected delivery date is available, pregnancy onset is estimated via the median duration of pregnancies with the respective outcome [16]. Live births are linked to mothers using an algorithm based on family insurance information [17].

### Study population and study design

We included all pregnancies ending in live births in GePaRD beginning between 01 January 2006 and 31 December 2019 in women aged 12–50 years at pregnancy onset with (i) at least 90 days of continuous insurance before the first day of pregnancy, (ii) continuous insurance during the whole duration of pregnancy, and (iii) exposure to RAAS-I or antihypertensives recommended during pregnancy in Germany, i.e., metoprolol or methyldopa (HYP) between day 140 and end of pregnancy (see definition below).

Children born from these pregnancies were followed up until (i) death, (ii) end of continuous insurance or (iii) end of study period, i.e., 31 December 2021, whichever occurred first.

### Exposure definition

Pregnancy was defined as exposed to RAAS-I/HYP in the critical time window for fetotoxicity, i.e., between day 140 and end of pregnancy if (i) a dispensed RAAS-I/HYP supply extended beyond day 140 and was followed by another dispensing before the end of pregnancy (i.e., mother was exposed on day 140 and continued use) or (ii) if there was no dispensing overlapping day 140 but RAAS-I/HYP were dispensed in the time period “day 140 to one week before the end of pregnancy” (i.e., the mother (re)started use on day 140 or later during pregnancy). The dispensing supply was estimated by the number of defined daily doses (DDDs) included in the dispensed packages. Exposure categories of interest were (i) exposure to RAAS-I in the critical time window and (ii) exposure to HYP but not to RAAS-I in the critical time window.

### Outcome definition

In a first step, we assessed whether a RAAS-I-related fetopathy was diagnosed in the first 180 days after birth. The following conditions were considered: (i) congenital hypoplasia and dysplasia and other specified or unspecified congenital malformation of the lung; (ii) Potter syndrome, kidney dysplasia, anuria and oliguria, severe kidney impairment, chronic kidney failure, dialysis or kidney transplant, cystic kidney disease or other (un)specified congenital malformations of the kidney; (iii) embolism and thrombosis of vena cava; (iv) contractures, congenital dis- or subluxation of hip, talipes equinovarus, arthrogryposis, other (un)specified congenital malformations of skull and face bones; and (v) sequalae of oligohydramnios. The underlying International Classification of Diseases, 10th modification, German version (ICD-10-GM) and Anatomical Therapeutic Chemical (ATC) codes and the algorithms used to define these conditions based on GePaRD are provided in Supplement 1.

In the next step, we assessed the long-term outcomes in children with fetopathy diagnoses in the first 180 days. Due to the small number of children with fetopathy, the long-term outcomes are described narratively.

In the final step, we assessed the occurrence of hypertension and diseases of the kidney system in children without fetopathy diagnoses in the first 180 days. Hypertension was defined as a diagnosis of hypertension or a dispensing of a diuretic, beta blocking agent, calcium channel blocker, ACE inhibitor, angiotensin II receptor blocker, renin-inhibitor, alpha-1-adrenoreceptor antagonist, methyldopa, minoxidil, or organic nitrate. Diseases of the kidney were defined as a diagnosis of anuria or oliguria, severe kidney impairment and kidney failure, chronic kidney disease stage 1–3, or procedure code for dialysis or kidney transplant. The underlying codes and algorithms used to define these conditions based on GePaRD are provided in Supplement 2.

### Further study variables

We assessed the following maternal characteristics: education level [18], age at onset of pregnancy, obesity status, use of antidiabetics and diuretics before and during the respective pregnancy, history of eclampsia or preeclampsia and occurrence of eclampsia or preeclampsia during the respective pregnancy as well as preterm birth. The underlying codes are provided in Supplement 3.

### Ethics statement

In Germany, the utilization of health insurance data for scientific research is regulated by the Code of Social Law. All involved health insurance providers as well as the German Federal Office for Social Security and the Senator for Health, Women and Consumer Protection in Bremen as their responsible authorities approved the use of GePaRD data for this study. Informed consent for studies based on claims data is required by law unless obtaining consent appears unacceptable and would bias results, which was the case in this study. According to the Ethics Committee of the University of Bremen, studies based on GePaRD are exempt from institutional review board review.

## Results

Overall, we identified 203 live born children exposed to RAAS-I during the critical time window (born from 199 pregnancies in 195 mothers) and 29,674 live born children exposed to HYP during the critical time window (born from 28,857 pregnancies in 26,442 mothers). Of the 203 RAAS-I-exposed children, 140 were exposed to ACE inhibitors, 61 to ARBs, and one to renin inhibitors, and one was exposed both to ACE inhibitors and ARBs (see Supplementary Fig. 1).

Mothers who used RAAS-I in the critical time window were older (in median 34 vs. 33 years) and less educated than mothers who used HYP. They also used antidiabetics and diuretics more frequently before and during pregnancy. Details are shown in Table 1.

**Table 1:** Maternal characteristics in pregnancies exposed to RAAS-I or HYP

	RAAS-I	HYP
	*N* = 199	*N* = 28,875
**Age at pregnancy onset**		
Median (25^th^ – 75^th^ percentile)	34.0 (31.0–37.0.0.0)	33.0 (29.0–36.0.0.0)
**Level of education (*n, %)***		
Higher education	72 (36.2)	13,946 (48.3)
(Basic) secondary degree	106 (53.3)	13,659 (47.3)
Degree unknown or no (formal) degree	21 (10.6)	1,270 (4.4)
**Comorbidity and comedication (***n***, %)**		
Obesity before or during pregnancy	106 (53.3)	14,747 (51.1)
Use of antidiabetics before pregnancy	21 (10.6)	1,580 (5.5)
Use of antidiabetics during pregnancy	26 (13.1)	2,951 (10.2)
Use of diuretics before pregnancy	59 (29.6)	3,273 (11.3)
Use of diuretics during pregnancy	60 (30.2)	541 (1.9)
History of preeclampsia	15 (7.5)	2,980 (10.3)
Preeclampsia during pregnancy	32 (16.1)	11,502 (39.8)
History of eclampsia	2 (1.0)	173 (0.6)
Eclampsia during pregnancy	3 (1.5)	384 (1.3)

Children in the RAAS-I exposure group were more often female and less often multiples than those in the HYP exposure group. There was no difference in median gestational age at birth between exposure groups (Table 2).

**Table 2:** Neonatal characteristics

	RAAS-I	HYP
	*N* = 203	*N* = 29,674
**Sex (***n***, %)**		
Females	111 (54.7)	14,243 (48.0)
**Multiples (***n***, %)**		
Yes	9 (4.4)	1,803 (6.1)
**Gestational age at birth (in days)**		
Median (25^th^ – 75^th^ percentile)	274.0 (263.0–280.0)	273.0 (263.0–280.0)
**Preterm (***n***, %)**		
Up to gestational week 33+6	10 (4.9)	1,597 (5.4)
Gestational week 34+0–36+6	31 (15.3)	3,824 (12.9)

### Live born children with RAAS-I-related fetopathy

Eight of 203 live born children (3.9%) exposed to RAAS-I during the critical time window had diagnoses consistent with RAAS-I-related fetopathy (only ACE inhibitors: one of 140 (0.7%), only ARBs: seven of 61 (11.5%), only renin inhibitors: zero of one, both ACE inhibitors and ARBs: zero of one), compared to 328 of 29,674 children (1.1%) with exposure to the recommended antihypertensives. Four children (2.0%) with RAAS-I exposure had congenital hypoplasia and dysplasia of the lung (ARBs: four of 61 (6.6%)) compared to 16 (0.1%) of the HYP-exposed ones. Kidney anomalies characteristic for RAAS-I-related fetopathy were diagnosed in five (2.5%) RAAS-I-exposed children (ARBs: four of 61 (6.5%), ACE inhibitors: one of 140 (0.7%)) and 84 (0.3%) HYP-exposed children. Embolism and thrombosis of the vena cava occurred in one (0.5%) child exposed to RAAS-I (ARBs: one of 61 (1.6%)), compared to ten (< 0.1%) children exposed to HYP. The ICD-10-GM diagnoses for the analyzed skeletal malformations and “consequences of oligohydramnios” were found with similar frequency in patients exposed to RAAS-I and HYP (0.5% vs. 0.6% and < 0.1% vs. 0.1%, see Table 3).

**Table 3:** ICD-10-GM diagnoses consistent with RAAS-I-related fetopathy in RAAS-I- and HYP-exposed infants (first 180 days of life)

	RAAS-I	HYP
	*N*= 203	*N* = 29,674
**ICD-10-GM diagnoses characteristic of RAAS-I-related fetopathy**	8 (3.9)	328 (1.1)
**Lung (n, %)**		
Congenital hypoplasia and dysplasia of lung (Q33.6)	4 (2.0)	16 (0.1)
Other (un)specified congenital malformations of lung (Q33.8 & Q33.9)	0 (0.0)	3 (0.0)
**Kidney (n, %)**		
Kidney anomalies	5 (2.5)	84 (0.3)
Potter-Syndrome (Q60.6) and kidney dysplasia (Q61.4)	2 (1.0)	18 (0.1)
Anuria and oliguria (R34)	0 (0.0)	24 (0.1)
Severe kidney impairment and chronic kidney failure^a^	4 (2.0)	31 (0.1)
Cystic kidney disease (Q61.3, Q61.5, Q61.8, Q61.9)	1 (0.5)	24 (0.1)
Other (un)specified congenital malformations of kidney (Q63.8 & Q63.9)	0 (0.0)	27 (0.1)
**Embolism and thrombosis of vena cava (I82.2) (n, %)**	1 (0.5)	10 (0.0)
**Skeletal (n, %)**		
Contractures, dis- or subluxation of the hip, talipes equinovarus or arthrogryposis	1 (0.5)	189 (0.6)
Among those multiples	0 (0.0)	13 (6.9)
Contractures (M24.5)	1 (0.5)	6 (0.0)
Among those multiples	0 (0.0)	1 (16.7)
Congenital dis- or subluxation of hip (Q65.0-Q65.5)	0 (0.0)	115 (0.4)
Among those multiples	0 (0.0)	4 (3.5)
Talipes equinovarus (Q66.0)	0 (0.0)	69 (0.2)
Among those multiples	0 (0.0)	8 (11.6)
Arthrogryposis (Q74.3)	0 (0.0)	2 (0.0)
Among those multiples	0 (0.0)	0 (0.0)
**Only other (un)specified congenital malformations of skull and face bones (Q75.8 & Q75.9)**	2 (1.0)	74 (0.2)
Thereof multiples	0 (0.0)	6 (8.1)
**Newborn affected by oligohydramnios (P01.2) (n, %)**	0 (0.0)	35 (0.1)

^a^N18.0, N18.4, N18.5, N18.84, P96.0, Z99.2 or procedure codes for dialysis or kidney transplantation

### Clinical long-term course of children with RAAS-I-related fetopathy

For the eight children with RAAS-I-related fetopathy, characteristics and details of the clinical course, postnatal and long term, are shown in Table 4 and Supplementary Table 1. All children were singletons, six were born prematurely (gestational age < 37 weeks). Four of eight newborns (infants 1–4) died within the first week of life. Three of these four were diagnosed with lung hypoplasia/dysplasia (infants 1–3) and three (infants 2–4) received kidney-related diagnoses (dysplasia, hypoplasia and/or congenital failure). The newborn who died without a diagnosis of lung hypoplasia/dysplasia (infant 4) was extremely premature (gestational age < 28 weeks) and was diagnosed with dysplasia of the kidneys with congenital kidney failure, respiratory failure, and multiple congenital malformations.

**Table 4:** Description of infants with fetopathy

	Exposure^1^	Delivery	Most relevant diagnoses during the first 180 days of life	Clinical course
**Infant 1**	ARB^2^	Moderately preterm^4^	Pulmonary hypoplasia and dysplasia with respiratory failure, drug-induced arterial hypotension.	Neonatal death in the first week of life.
**Infant 2**	ARB^2^	Late preterm^5^	Hypoplasia of the kidneys, pulmonary hypoplasia and dysplasia with respiratory failure, congenital malformations of skull and face bones, esophageal atresia.	Neonatal death in the first week of life.
**Infant 3**	ARB^2^	Late preterm^5^	Congenital kidney failure, pulmonary hypoplasia and dysplasia with respiratory failure, arterial hypotension.	Neonatal death in the first week of life.
**Infant 4**	ARB^2^	Extremely preterm^6^	Dysplasia of the kidneys with congenital kidney failure, respiratory distress syndrome of the newborn with respiratory failure, multiple congenital malformations, prematurity-related conditions.	Neonatal death in the first week of life.
**Infant 5**	ACE Inhibitor^3^	Full term^6^	Dysplasia of the kidneys, polycystic kidney^5^.	Follow-up: 3.2 years. No kidney replacement therapy, no arterial hypertension. Hospitalizations and outpatient treatment for urinary tract infections and tubulointerstitial nephritis. Outpatient diagnosis of unilateral kidney agenesis.
**Infant 6**	ARB^2^	Late preterm^4^	Congenital kidney insufficiency, respiratory distress syndrome of the newborn with respiratory failure, congenital malformation of tricuspid valve, congenital mitral insufficiency.	Follow-up: 7.4 years. Chronic kidney disease stage 1–2 with regular monitoring and hospitalization. No arterial hypertension.
**Infant 7**	ARB^2^	Full term^7^	Respiratory distress of the newborn, joint contractures in several places, congenital malformations of the upper extremity and knee, other congenital musculoskeletal deformities, congenital hydrocephalus, muscle hypotonia and disturbance of temperature regulation of the newborn.	Follow-up: 13.7 years. No kidney replacement therapy, no kidney-related diagnoses and no arterial hypertension.
**Infant 8**	ARB^2^	Moderately preterm^4^	Congenital kidney failure, pulmonary hypoplasia and dysplasia, pneumothorax and bronchopulmonary dysplasia with respiratory distress syndrome of the newborn and cardiac insufficiency, thrombosis/embolism of vena cava, congenital deformity of feet.	Follow-up: 11 months. Several peritoneal dialyses, most of the time hospitalized.

^1^In the critical time window for fetotoxicity, i.e., between day 140 and end of the pregnancy

^2^Angiotensin II receptor blocker

^3^Angiotensin-converting enzyme inhibitor

^4^Moderately preterm: gestational week (GW) 32+0–33+6;

^5^ Late preterm: GW 34+0–36+6;

^6^ Extremely preterm: before GW 28+0; ^7^Full term: GW 37+0–40+6

Of the four surviving children (infants 5–8), one was diagnosed with lung hypoplasia/dysplasia (infant 8) followed by diagnoses of congenital kidney failure and vena cava thrombosis at 8 weeks of age. During the 11 months of follow-up, the infant was hospitalized most of the time and received several peritoneal dialyses. The other three children (infants 5–7) required no kidney replacement therapy. However, infant 5 was diagnosed with dysplastic and polycystic kidneys and was repeatedly treated and hospitalized for urinary tract infections and tubulointerstitial nephritis during 3.2 years of follow-up. Infant 6 was diagnosed with chronic kidney disease stage 1, intermittently also stage 2, and was regularly monitored and hospitalized during 7.4 years of follow-up. Only infant 7 did not receive kidney-related diagnoses either at birth or during 13.7 years of follow-up.

### Long-term outcomes of children without fetopathy

In children without neonatal symptoms of fetopathy (RAAS-I-exposed group; *n* = 195; HYP-exposed group: *n *= 29,346), median follow-up was 4.0 years with 10.8% (*n* = 21) of RAAS-I- and 11.6% (*n* = 3416) of HYP-exposed children followed up for more than 10 years (more than 7 years: 27.2% of RAAS-I- and 26.9% of HYP-exposed children, see Table 5). Codes indicating kidney disease associated with kidney insufficiency were found in no child with RAAS-I exposure and in 16 children (0.1%) exposed to HYP. However, five of the 195 RAAS-I-exposed children (2.6%) were diagnosed with urinary malformations after the first 6 months of life: one each with kidney dysplasia, kidney hypoplasia and lobulated, fused or horseshoe kidney, two with congenital ureteral atresia/stenosis. Among HYP-exposed children, 419 (1.4%) were diagnosed with a urinary malformation, with 32 (0.1%) with kidney dysplasia and 54 (0.2%) with kidney hypoplasia (see also Supplementary Fig. 1). In one (20.0%) of the RAAS-I-exposed and 49 (11.7%) of the HYP-exposed children with urinary malformations, the mother had used antidiabetics before or during the pregnancy. A total of three children with RAAS-I exposure (1.5%) were diagnosed with hypertension or received antihypertensive prescriptions: One child was diagnosed and hospitalized with hypertension secondary to kidney disorders (ICD-10-GM: I15.10) and hypoplastic left heart syndrome (Q23.4) in the first year of life, two were prescribed antihypertensives at the ages of 1 and 9. The latter two were both diagnosed with combined atrial and ventricular septal defects. Among children exposed to HYP, 176 children (0.6%) had hypertension (ICD-10-GM code or antihypertensive medication).

**Table 5:** Characteristics and long-term outcomes of children without RAAS-I-related fetopathy

	RAAS-I	HYP
Characteristics of children	*N* = 195	*N*=29,346
Follow-up in years		
Median (25^th^ – 75^th^ percentile)	4.0 (1.6–7.3)	4.0 (1.9–7.3)
More than seven years	53 (27.2%)	7,904 (26.9%)
More than 10 years	21 (10.8%)	3,416 (11.6%)
Sex		
Female	106 (54.4)	14,078 (48.0)
Maternal use of thiazide diuretics		
Before pregnancy onset	15 (7.7)	1,022 (3.5)
During pregnancy	4 (2.1)	121 (0.4)
Before or during pregnancy	17 (8.7)	1,056 (3.6)
**Chronic kidney dysfunction or oliguria/anuria (*n*, %)**		
Anuria or oliguria (R34)	0 (0.0)	11 (0.0)
Severe kidney impairment and chronic kidney failure^a^	0 (0.0)	2 (0.0)
Dialysis	0 (0.0)	4 (0.0)
Kidney transplant	0 (0.0)	0 (0.0)
Chronic kidney disease (CKD) stage 1–3^b^	0 (0.0)	17 (0.1)
**Hypertension (*n*, %)**		
Diagnosis code(s)	1 (0.5)	53 (0.2)
Use of antihypertensives		
Any	2 (1.0)	140 (0.5)
Diuretics	1 (0.5)	47 (0.2)
Beta blockers	0 (0.0)	72 (0.2)
Calcium channel blockers	0 (0.0)	6 (0.0)
ACE inhibitors	1 (0.5)	25 (0.1)
Angiotensin II receptor blockers	0 (0.0)	7 (0.0)
Renin-inhibitors	0 (0.0)	0 (0.0)
Alpha-1-adrenoreceptor antagonists	0 (0.0)	0 (0.0)
Methyldopa	0 (0.0)	3 (0.0)
Minoxidil	0 (0.0)	0 (0.0)
Organic nitrates	0 (0.0)	1 (0.0)
Clonidine	0 (0.0)	1 (0.0)
Diagnosis code(s) and antihypertensives	0 (0.0)	17 (0.1)
Only diagnosis code(s)	1 (0.5)	36 (0.1)
Only antihypertensives	2 (1.0)	123 (0.4)
**Urinary malformations** (Q60 – Q64)^c^ (n, %)	5 (2.6%)	419 (1.4%)
Kidney dysplasia and kidney hypoplasia	2 (1.0%)	86 (0.3%)

^a^N18.0, N18.4, N18.5, N18.84, P96.0, Z99.2

^b^I12.0, I13.1, I13.2, N18.1, N18.2, N18.3, N18.8, N18.80, N18.81, N18.82, N18.83, N18.89, N18.89, N18.9, N19

^c^Coded only during follow-up, i.e., more than 180 days after birth

## Discussion

We analyzed postnatal and long-term clinical outcomes in 203 live born infants exposed to RAAS-I in the second half of pregnancy. This is the largest RAAS-I-exposed cohort analyzed to date and the only one with long-term data on both fetopathy and non-fetopathy cases. For comparison, the same outcomes were analyzed in a cohort of 29,346 live births exposed to the antihypertensive drugs alpha-methyldopa or metoprolol, which are recommended for use during pregnancy in Germany.

### Occurrence of fetopathy

Occurrence of fetopathy depends on exposure time and duration during pregnancy. Its frequency may therefore vary between different study cohorts. In our study, RAAS-I-related fetopathy occurred in 0.7% (*n* = 1/141) of ACE-I-exposed and 11.3% (*n* = 7/61) of ARB-exposed live births. This marked difference between RAAS-I inhibitors has already been observed in a systematic review of published cases with RAAS-I exposure [19] and in a study by Weber-Schoendorfer et al. [4]. The latter included 55 prospectively ascertained pregnancies; among those, 3.2% (*n* = 1/31) of ACE-I-exposed and 29.2% (*n* = 7/24) of ARB-exposed fetuses and newborns suffered from fetopathy. The overall higher frequency of fetopathy in comparison to our study is likely partly because pregnancy losses were included in this former study. We only included live born children, as the focus of our study was on postnatal and long-term outcomes. Since the occurrence of RAAS-I-related fetopathy can lead to intrauterine fetal death (IUFD) and termination of pregnancy due to fetal anomaly (TOPFA), excluding these pregnancies may reduce the proportion of affected fetuses. Another methodological difference is even more important: Fetopathy was identified based on neonatal symptoms in our study. By contrast, Weber-Schoendorfer et al. also classified oligohydramnios during pregnancy as a symptom of fetopathy even if no evidence of fetopathy was observed in the newborn. In their prospective cohort, there were five fetopathy cases in which oligohydramnios was the only clinical manifestation of fetopathy. In some of these pregnancies, oligohydramnios was reversible after discontinuation of RAAS-I. If cases exclusively presenting with oligohydramnios had not been classified as fetopathy, there would have been no fetopathy cases among those exposed to ACE-I (*n* = 0/31) and three cases among those exposed to ARB (3/24). In turn, the non-fetopathy cohort in our study, defined by the absence of postpartum fetopathy symptoms, can be expected to include children with transient impaired fetal kidney function during pregnancy and consecutive (reversible) oligohydramnios. Thus, considering the methodological differences between the studies, the different results on fetopathy rates can be reconciled quite consistently.

### Postnatal and long-term outcome

Our study is the first to follow up a cohort of 203 infants with in utero exposure to RAAS-I beyond the postnatal period. Furthermore, outcome rates could be compared with a disease control group of approximately 29,000 children with in utero exposure to recommended antihypertensives. In our RAAS-I exposed cohort, four of eight newborns with fetopathy died in the first week of life. A similarly high lethality in newborns with fetopathy has been reported in case series: Neonatal deaths in the first week of life were reported in five out of 11 live births with fetopathy by Spaggiari et al. [12] and in four out of seven by Hünseler et al. [10]. Weber-Schoendorfer et al. [4] reported a lower lethality: Out of 54 live births with fetopathy (prospectively and retrospectively ascertained cases), seven died in the neonatal period and three more died in the first year of life.

Three out of four surviving children with fetopathy in our cohort were diagnosed with congenital kidney insufficiency or dysplastic and polycystic kidneys (follow-up eleven months, 3.2, and 7.4 years), only one did not receive any kidney-related diagnoses (follow-up 13.7 years). None of these four children was diagnosed with arterial hypertension. Several case series also described postnatal and long-term courses of children with RAAS-I-related fetopathy [10–12], most of them from highly selective clinical samples, e.g., pediatric nephrology or other specialized centers. The case series by Hünseler et al. [10] reported on a 1-year follow-up of three surviving infants with fetopathy. All were diagnosed with kidney insufficiency, two with arterial hypertension. Spaggiari et al. [12] followed up six surviving children with fetopathy for between 1 and 9 years. Only one out of six (2 years of follow-up) had normal kidney function, five developed chronic kidney insufficiency. Nadeem et al. [11] described the clinical course of 24 RAAS-I-exposed children with affected kidneys, mean age at last follow-up was 4.7 years. Of 17 children with exposure after the first trimester, eight needed chronic kidney replacement therapy and six developed neonatal hypertension. In summary, all case series described persistent kidney impairment in a high proportion of surviving children with fetopathy like our study cohort. However, concomitant arterial hypertension, as frequently observed in the case series, was not diagnosed in the four surviving children with fetopathy in our cohort.

In 195 of 203 RAAS-I-exposed infants, there were no diagnoses indicating RAAS-I-related fetopathy in the first 6 months of life. However, two children without fetopathy were later diagnosed with kidney dysplasia and kidney hypoplasia (leading to secondary hypertension), respectively. These anomalies may have been caused by fetal exposure to RAAS-I. Two other children were prescribed antihypertensives at the ages of 1 and 9 years. As they were also diagnosed with major cardiac malformations (combined atrial and ventricular septal defect and hypoplastic left heart syndrome), the antihypertensive medication may have been prescribed in the context of these diagnoses. In summary, only a few children not diagnosed with fetopathy at birth were later diagnosed with kidney anomalies or arterial hypertension that could be due to fetal RAAS-I exposure. All these children had congenital heart and/or kidney defects that could have been detected by ultrasound in the newborn. Our median follow-up was 4 years (25th–75th percentile, 1.6–7.3); just over a tenth of the children were followed for more than 10 years, and just over a quarter for more than 7 years. Possible consequences of fetal RAAS-I exposure such as chronic kidney disease and arterial hypertension do not necessarily manifest in the first years of life [8]. Therefore, the follow-up period may not have been long enough to capture all children with clinical sequelae of RAAS-I exposure.

### Differences between exposure groups

Pregnancies exposed to RAAS-I differed from pregnancies exposed to metoprolol or methyldopa regarding maternal use of antidiabetics and diuretics. These medications were more prevalent among women with RAAS-I exposure, which is consistent with RAAS-I being used to treat early-stage diabetic kidney disease, and may also reflect the wide availability of combination products containing RAAS-I and hydrochlorothiazide. Additionally, as methyldopa is primarily used to treat hypertension during pregnancy, it is reasonable to assume that healthcare providers who prescribe methyldopa adjust the medication for pregnancy and therefore rarely prescribe diuretics.

As hypertension during pregnancy, including when it occurs in the context of preeclampsia, is one of the indications of HYP during pregnancy, it is plausible that the proportion of pregnancies with preeclampsia was higher in the HYP exposed than in the RAAS-I exposed pregnancies.

To examine the impact of these differences, we stratified Table 5 by maternal use of antidiabetics and diuretics and the occurrence of preeclampsia (see Supplementary Table 2). Overall, stratified results were like those of the unstratified analysis, indicating that the observed higher proportion of hypertension and urinary malformations in RAAS-I exposed children compared to HYP exposed children cannot be explained by these differences alone.

The number of RAAS-I exposed children with diagnoses indicating the long-term outcomes of interest was too small (*n* = 3 for hypertension, *n* = 5 for urinary malformations) to build a multivariable model estimating the impact of RAAS-I exposure on the respective outcome, adjusted for potential risk factors and confounders. Adjusting for the potential risk factors “use of antidiabetics” or “use of diuretics” would most likely shift the risk ratio of RAAS-I exposure compared to exposure to HYP towards one, i.e., to no effect.

### Strengths and limitations

The strengths of our study are the size of the study population and the population-based approach (i.e., no hospital-based recruitment as in other studies), the lack of non-responder or recall bias, the completeness of dispensing data, and the long study period allowing us to examine long-term outcomes. We also used validated algorithms to identify pregnancies and to estimate pregnancy onset based on the expected delivery date.

As is the case for all studies based on secondary data, this analysis has limitations, mainly inherent to the data source. First, claims data are collected for billing purposes. Therefore, the coding of diagnoses is not always optimal for scientific purposes. Second, although our cohort is the largest to date comprising children with in utero exposure to RAAS-I (*n *= 203), cohort size is still limited and we did not adjust for potentially confounding factors for the occurrence of oligohydramnios and fetopathy such as concomitant exposure to diuretics. Furthermore, there is no single ICD-10-GM code specific to RAAS-I-related fetopathy. As a result, even in the HYP-exposed cohort, there is a small percentage of children with diagnoses consistent with RAAS-I-induced fetopathy. In the first 180 days of life, for example, 2.5% of RAAS-I-exposed newborns and 0.3% of HYP-exposed ones were diagnosed with ICD-10-GM codes for kidney anomalies consistent with RAAS-I-related fetopathy.

### Clinical implications

RAAS-I-related fetopathy can develop into a life-threatening condition with high lethality, particularly in the first week of life. Although RAAS-I-related fetopathy occurs with both ACE-I and ARB exposure, our results support prior findings that the risk is markedly higher for ARBs. RAAS-I should be used with caution in women of childbearing age, especially considering that pre-pregnancy counselling regarding fetotoxic risks may not be optimal [20] and that in Germany, similar to other countries, at least one-third of pregnancies are unplanned [21, 22]. If the use of RAAS-I cannot be avoided in women of reproductive age, ACE-I should be preferred to ARB if possible, and women should be advised that RAAS-I must be discontinued prior to conception or, at the latest, as soon as pregnancy is detected. If exposure has occurred in the second half of pregnancy, careful monitoring of the pregnancy, including amniotic fluid analysis and the newborn child is essential. In our cohort, most exposed children without fetopathy diagnoses at birth did not develop chronic kidney disease or arterial hypertension during the follow-up period. Nevertheless, ultrasound examination of the kidneys may be indicated in exposed newborns without clinical symptoms of fetopathy to detect sequelae of fetal RAAS-I exposure requiring further clinical monitoring.

## Supplementary Information

Below is the link to the electronic supplementary material.

Graphical abstract (PPTX 95.2 KB)

ESM 1 (DOCX 137 KB)

## Data Availability

As we are not the owners of the data, we are not legally entitled to grant access to the data of the German Pharmacoepidemiological Research Database. In accordance with German data protection regulations, access to the data is granted only to BIPS employees on the BIPS premises and in the context of approved research projects. Third parties may only access the data in cooperation with BIPS and after signing an agreement for guest researchers at BIPS.
