# Supplementary material for: Postnatal and long-term outcomes after in utero exposure to RAAS inhibitors: cohort study based on German claims data
Source: Pediatr Nephrol. 2025 Dec 17;41(5):1387–97. doi: 10.1007/s00467-025-07101-9 (PMC13009111; doi:10.1007/s00467-025-07101-9)
Supplement: Supplementary file 1 — Graphical abstract (PPTX 95.2 KB) [file 467_2025_7101_MOESM1_ESM.pptx]

## Slide 1
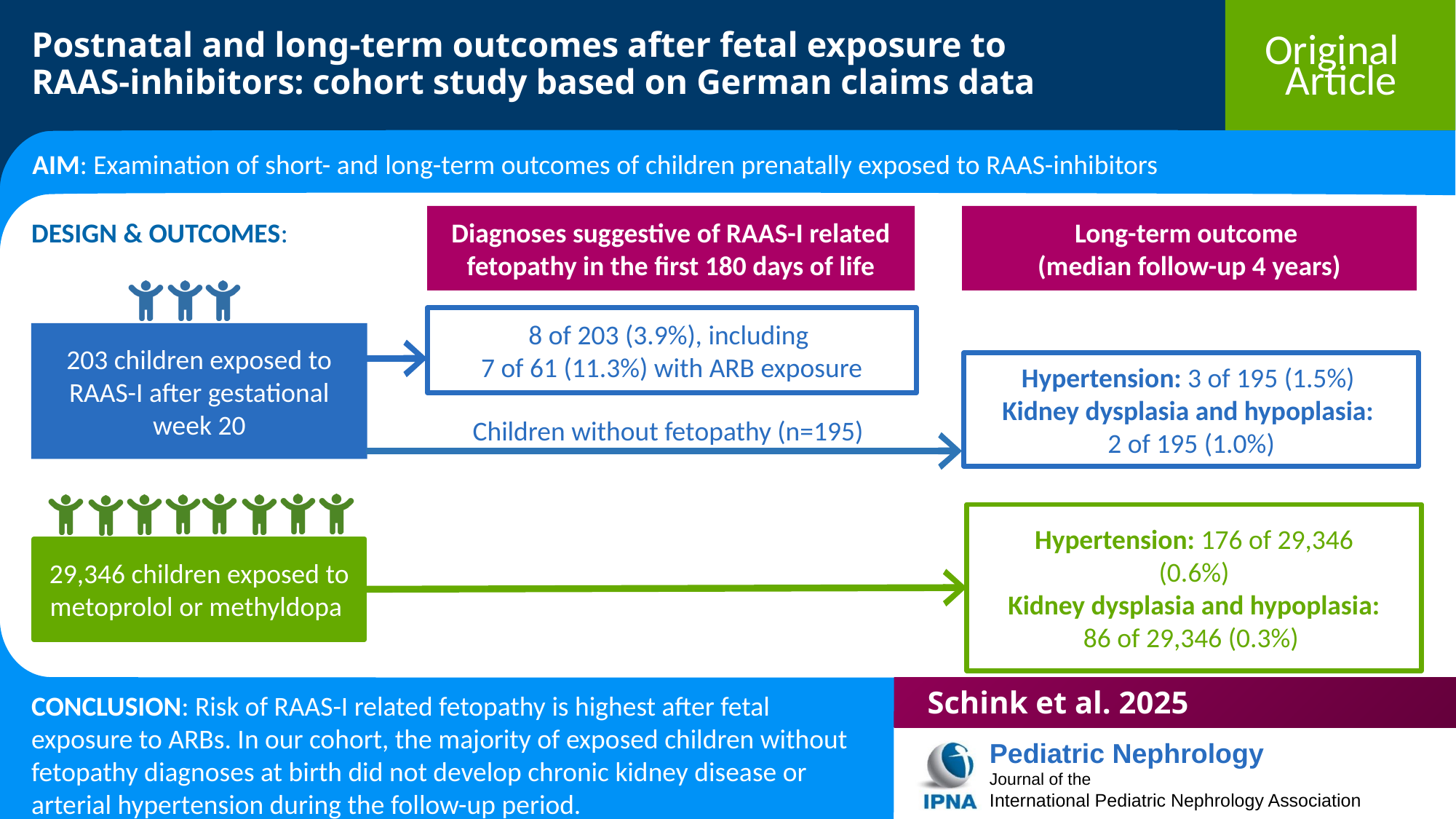

Postnatal and long-term outcomes after fetal exposure to
RAAS-inhibitors: cohort study based on German claims data
AIM: Examination of short- and long-term outcomes of children prenatally exposed to RAAS-inhibitors
Diagnoses suggestive of RAAS-I related fetopathy in the first 180 days of life
Long-term outcome
(median follow-up 4 years)
DESIGN & OUTCOMES:
8 of 203 (3.9%), including
7 of 61 (11.3%) with ARB exposure
203 children exposed to RAAS-I after gestational week 20
Hypertension: 3 of 195 (1.5%)
Kidney dysplasia and hypoplasia:
2 of 195 (1.0%)
Children without fetopathy (n=195)
Hypertension: 176 of 29,346
(0.6%)
Kidney dysplasia and hypoplasia:
86 of 29,346 (0.3%)
29,346 children exposed to metoprolol or methyldopa
Schink et al. 2025
CONCLUSION: Risk of RAAS-I related fetopathy is highest after fetal exposure to ARBs. In our cohort, the majority of exposed children without fetopathy diagnoses at birth did not develop chronic kidney disease or arterial hypertension during the follow-up period.
