# Supplementary material for: Postnatal and long-term outcomes after in utero exposure to RAAS inhibitors: cohort study based on German claims data
Source: Pediatr Nephrol. 2025 Dec 17;41(5):1387–97. doi: 10.1007/s00467-025-07101-9 (PMC13009111; doi:10.1007/s00467-025-07101-9)
Supplement: Supplementary file 2 — (DOCX 137 KB) [file 467_2025_7101_MOESM2_ESM.docx]

**Postnatal and long-term outcomes after in utero exposure to RAAS-inhibitors: cohort study based on German claims data**

Tania Schink^1^, Malte Braitmaier^2^, Katarina Dathe^3^, Ulrike Haug^1,4^, Christof Schaefer^3^, Kathrin Thöne^5^, Marlies Onken^3^

^1^ Department of Clinical Epidemiology, Leibniz Institute for Prevention Research and Epidemiology – BIPS, Bremen, Germany

^2^ Department Statistical Methods in Epidemiology, Leibniz Institute for Prevention Research and Epidemiology - BIPS, Bremen, Germany

^3^ Charité – Universitätsmedizin Berlin, corporate member of Freie Universität Berlin, Humboldt-Universität zu Berlin, and Berlin Institute of Health, Institut für Klinische Pharmakologie und Toxikologie, Pharmakovigilanz- und Beratungszentrum für Embryonaltoxikologie, Berlin, Germany

^4^ Faculty of Human and Health Sciences, University of Bremen, Bremen, Germany

^5^ Techniker Krankenkasse, Hamburg, Germany

Corresponding author

Tania Schink, [schink@leibniz-bips.de](mailto:schink@leibniz-bips.de)

**Supplement 1. Definition and assessment of fetotoxic events**

Fetotoxic events were classified in 5 groups with 12 subgroups based on ICD-10 diagnoses:

- “Lung” with 2 subgroups
  - Congenital hypoplasia and dysplasia of the lung (Q33.6),
  - Other (un)specified congenital malformations of lung (Q33.8 & Q33.9)
- “Renal system” with 5 subgroups
  - Potter-Syndrome (Q60.6) and renal dysplasia (Q61.4)
  - Anuria und oliguria (R34),
  - Severe renal impairment and chronic kidney failure (N180, N184, N185, N1884, P960, Z992 or procedure codes for dialysis or renal transplant
  - Cystic kidney disease (Q61.3, Q61.5, Q61.8, Q61.9)
  - Other (un) specified congenital malformations of kidney (Q63.8 & Q63.9)
- Embolism and thrombosis of vena cava (I82.2)
- “Skeletal anomalies” with 5 subgroups
  - Contractures (M24.5)
  - Congenital dis- or subluxation of hip (Q65.0-Q65.5)
  - Talipes equinovarus (Q66.0)
  - Arthrogryposis (Q74.3)
  - Only other (un)specified congenital malformations of skull and face bones (Q75.8 & Q75.9)
- Newborn affected by oligohydramnios (P01.2)

Within each (sub)group, two outpatient diagnoses coded as certain or one hospital diagnosis (main discharge or secondary diagnosis) were required. For “severe renal impairment and chronic kidney failure” two outpatient diagnoses, one hospital diagnosis (main discharge or secondary diagnosis), one procedure code for kidney transplantation, two procedure codes for dialysis or one outpatient diagnosis with one procedure code for dialysis were required.

**Supplement 2. Definition and assessment of long-term-outcomes**

Long-term outcomes of interest were assessed using the following International Classification of Diseases, 10th modification, German version (ICD-10-GM) or Anatomical Therapeutic Chemical (ATC) codes:

- Hypertension, defined as a diagnosis of hypertension (I10-I15) or a dispensing of a diuretic (C03, C07C, C07D, C08G, C09BA, C09DA), beta blocking agent (C07), calcium channel blocker (C08), ACE inhibitor (C09A, C09B), angiotensin II receptor blocker (C09C, C09D), renin-inhibitor (C09XA), Alpha-1-adrenoreceptor antagonist (C02CA), methyldopa (C02AB), minoxidil (C02DC01), or organic nitrate (C01DA)

Two outpatient diagnoses coded as certain or one hospital diagnosis or one dispensing were required

- Diseases of the renal system, defined as a diagnosis of anuria or oliguria (R34), severe renal impairment and kidney failure (N180, N184, N185, N1884, P960, Z992), chronic kidney disease stage 1 – 3 (I120, I131, I132, N181, N182, N183, N188, N1880, N1881, N1882, N1883, N1889, N1889, N189, N19) a procedure code for dialysis or a procedure code for renal transplant. Two outpatient diagnoses coded as certain or one procedure code or one hospital diagnosis were required.

**Supplement 3. Assessment of further study variables**

- Obesity of the mother (E65, E66, R635) before or during the pregnancy: outpatient diagnosis coded as certain or hospital main or secondary diagnosis
- Use of antidiabetics (A10) before or during the pregnancy
- Use of diuretics (C03, C07C, C07D, C08G, C09BA, C09DA) before or during the pregnancy
- History of eclampsia or preeclampsia of the mother (O11, O14, O15): outpatient diagnosis coded as certain or hospital main or secondary diagnosis
- Eclampsia or preeclampsia of the mother (O11, O14, O15) during the pregnancy: outpatient diagnosis coded as certain or hospital main or secondary diagnosis

**Supplementary Table 1. Characteristics and clinical diagnoses in infants with fetopathy**

| **Infant, neonatal sex, delivery,**  **birth weight (BW)** | **Exposure** | **Most relevant clinical diagnoses (underlying ICD-10 codes) during the first 180 days of life** | **Clinical course** |
| --- | --- | --- | --- |
| Infant 1, female,  moderately preterm^1^,  BW between 75% and 90% percentile | Angiotensin II receptor blocker | - Pulmonary hypoplasia and dysplasia with respiratory failure (Q33.6, P22.0, P22.8, P28.5, P29.2, P29.3, P29.8, I27.0) - Drug-induced arterial hypotension (I95.2) | Neonatal death in the first week of life. |
| Infant 2, female,  late preterm^2^,  BW between 75% and 90% percentile | Angiotensin II receptor blocker | - Hypoplasia of the kidneys (Q60.4) - Pulmonary hypoplasia and dysplasia with respiratory failure  (Q33.6, P28.5) - Congenital malformations of skull and face bones (Q75.8, Q86.88) - Esophageal atresia (Q39.0) | Neonatal death in the first week of life. |
| Infant 3, male,  late preterm^2^, BW < 3 % percentile | Angiotensin II receptor blocker | - Congenital kidney failure (P96.0) - Pulmonary hypoplasia and dysplasia with respiratory failure (Q33.6, Q33.8, P21.0, P29.3, P28.5, P04.1) - Arterial hypotension (I95.9) | Neonatal death in the first week of life. |
| Infant 4, male,  extremely preterm^3^,  BW between 10% and 25% percentile | Angiotensin II receptor blocker | - Dysplasia of the kidneys with congenital kidney failure  (Q61.4, P96.0, N17.99) - Respiratory distress syndrome of the newborn with respiratory failure (P22.0, P28.5, J81) - Multiple congenital malformations (malformations of larynx and stomach, absence/atresia/stenosis of small intestine and anus, malformation of genital organs, atresia/stenosis of urethra and bladder neck with congenital megaureter, atrial septal defect). (Q31.8, Q40.2, Q41.9, Q42.3, K56.7, Q55.9, Q64.3, Q62.2, Q21.1) - Prematurity-related conditions (patent ductus arteriosus, anemia) (Q25.0, P61.2, P07.2) | Neonatal death in the first week of life. |
| Infant 5, female,  full term^4^,  BW between 25% and 50% percentile | Angiotensin-converting-enzyme (ACE) inhibitor | - Dysplasia of the kidneys, polycystic kidney^5^ (Q61.4, Q61.3) | No kidney replacement therapy, no arterial hypertension. Repeated hospitalizations and outpatient treatment for urinary tract infections and tubulointerstitial nephritis during 3.2 years of follow-up. |
| Infant 6, female,  late preterm^2^,  BW between 10% and 25% percentile | Angiotensin II receptor blocker | - Congenital kidney insufficiency (P96.0) - Respiratory distress syndrome of the newborn with respiratory failure (P22.0, P28.5) - Congenital malformation of tricuspid valve, congenital mitral insufficiency (Q22.8, Q23.3) | Chronic kidney disease stage 1–2 with regular monitoring and hospitalization during the follow-up period of 7.4 years. No arterial hypertension. |
| Infant 7, female,  full term^4^,  BW between 75% and 90% percentile | Angiotensin II receptor blocker | - Respiratory distress of the newborn (P22.8) - Joint contractures in several places, congenital malformations of the upper extremity and knee, other congenital musculoskeletal deformities (M24.50, Q74.0, Q74.1, Q68.8) - Congenital hydrocephalus, muscle hypotonia and disturbance of temperature regulation of the newborn (Q03.8, P94.2, P81.9) | No kidney replacement therapy, no kidney-related diagnoses and no arterial hypertension during the follow-up period of 13.7 years. |
| Infant 8, male, moderately preterm^1^,  BW between 10% and 25% percentile | Angiotensin II receptor blocker | - Congenital kidney failure, toxic nephropathy (P96.0, N19, I12.00, N14.4) - Pulmonary hypoplasia and dysplasia, pneumothorax and bronchopulmonary dysplasia with respiratory distress syndrome of the newborn and cardiac insufficiency  (Q33.6, P22.0, P25.1, P27.1, P28.4, I50.9) - Thrombosis/embolism of vena cava (I82.2) - Congenital deformity of feet (Q66.8) | Several peritoneal dialyses and most of the time hospitalized during the follow-up period of 11 months. |

**Supplementary Table 2.** **Stratification of Table 5:** Characteristics and long-term outcomes of children without RAAS-I-related fetopathy by occurrence of potential confounders maternal use of antidiabetics, maternal use of diuretics and preeclampsia before or during the respective pregnancy.

|  | **No maternal use of antidiabetics** | | **Maternal use of antidiabetics** | |
| --- | --- | --- | --- | --- |
|  | **RAAS-I** | **HYP** | **RAAS-I** | **HYP** |
|  | **N = 167** | **N = 25,885** | **N = 28** | **N = 3,461** |
| Follow-up in days |  |  |  |  |
| Median (Q1 – Q3) | 1,470.0 (598.0-2,801.0) | 1,483.0 (689.0-2,739.0) | 1,227.0 (484.5-1,838.0) | 1,234.0 (588.0-2,314.0) |
| Sex |  |  |  |  |
| Females | 91 (54.5%) | 12,440 (48.1%) | 15 (53.6 %) | 1,638 (47.3%) |
| Renal disease | 0 | 0 | 0 | 0 |
| Hypertension | 2 (1.2%) | 152 (0.6%) | 1 (3.6%) | 24 (0.7%) |
| Urinary malformations | 4 (2.4%) | 370 (1.4%) | 1 (3.6%) | 49 (1.4%) |
|  | **No maternal use of diuretics** | | **Maternal use of diuretics** | |
|  | **RAAS-I** | **HYP** | **RAAS-I** | **HYP** |
|  | **N = 121** | **N = 25,926** | **N = 74** | **N = 3,420** |
| Follow-up in days |  |  |  |  |
| Median (Q1 – Q3) | 1,594.0 (664.0-2,814.0) | 1,465.5 (679.0-2,714.0) | 1,260.0 (556.0-2,388.0) | 1,371.5 (646.0-2,428.0) |
| Sex |  |  |  |  |
| Females | 71 (58.7%) | 12,466 (48.1%) | 35 (47.3%) | 1,612 (47.1%) |
| Renal disease | 0 | 0 | 0 | 0 |
| Hypertension | 1 (0.8%) | 153 (0.6%) | 2 (2.7%) | 23 (0.7%) |
| Urinary malformations | 2 (1.7%) | 363 (1.4%) | 3 (4.1%) | 56 (1.6%) |
|  | **No preeclampsia** | | **Preeclampsia** | |
|  | **RAAS-I** | **HYP** | **RAAS-I** | **HYP** |
|  | **N = 162** | **N = 17,662** | **N = 33** | **N = 11,724** |
| Follow-up in days |  |  |  |  |
| Median (Q1 – Q3) | 1,330.0 (569.0-2,637.0) | 1,399.0 (645.0-2,614.0) | 1,997.0 (864.0-2,814.0) | 1,540.0 (722.0-2,784.0) |
| Sex |  |  |  |  |
| Females | 88 (54.3%) | 8,449 (47.9%) | 18 (54.5%) | 5,629 (48.0%) |
| Renal disease | 0 | 0 | 0 | 0 |
| Hypertension | 3 (1.8%) | 92 (0.5%) | 0 | 84 (0.7%) |
| Urinary malformations | 5 (3.1%) | 231 (1.3%) | 1 (3.0%) | 112 (1.0%) |


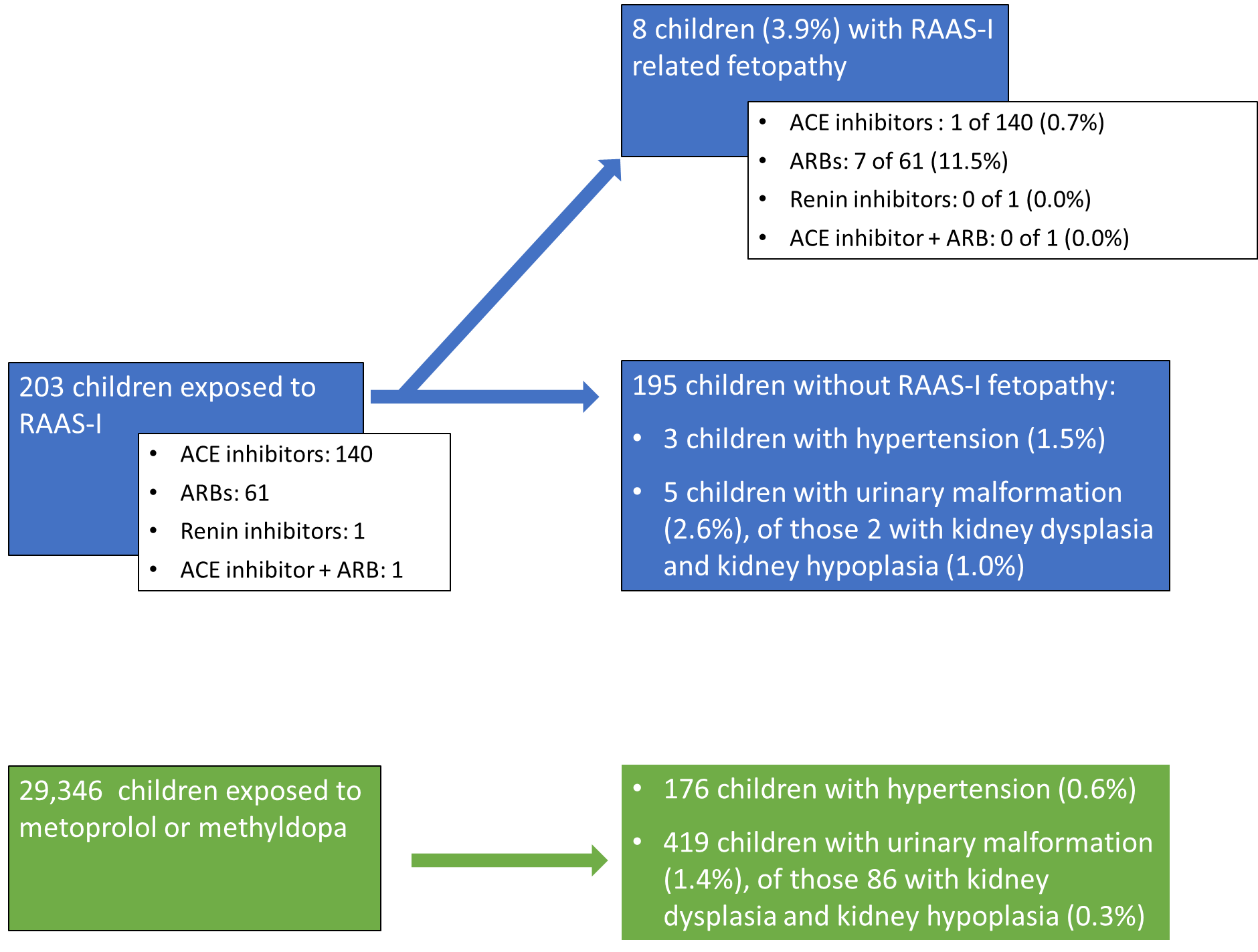


**Supplementary Figure 1. Flowchart**

RAAS-I, inhibitors of the renin-angiotensin-aldosterone system; ACE, angiotensin-converting-enzyme; ARB, angiotensin II receptor blockers
